# Potential Health Benefits of Curcumin on Female Reproductive Disorders: A Review

**DOI:** 10.3390/nu13093126

**Published:** 2021-09-07

**Authors:** Datu Agasi Mohd Kamal, Norizam Salamt, Allia Najmie Muhammad Yusuf, Mohd Izhar Ariff Mohd Kashim, Mohd Helmy Mokhtar

**Affiliations:** 1 Department of Physiology, Faculty of Medicine, Universiti Kebangsaan Malaysia, Kuala Lumpur 56000, Malaysia; agasi.mk@ums.edu.my (D.A.M.K.); norizam_salamt@ukm.edu.my (N.S.); 2 Department of Biomedical Sciences, Faculty of Medicine and Health Sciences, Universiti Malaysia Sabah, Kota Kinabalu 88400, Malaysia; allia.najmie@ums.edu.my; 3 Centre of Shariah, Faculty of Islamic Studies, Universiti Kebangsaan Malaysia, Bangi 43600, Malaysia; izhar.ukm@gmail.com; 4 Insitute of Islam Hadhari, Universiti Kebangsaan Malaysia, Bangi 43600, Malaysia

**Keywords:** *Curcuma longa*, therapeutic potential, polycystic ovary syndrome, endometriosis, ovarian diseases

## Abstract

Curcumin is one of the main polyphenolic compounds in the turmeric rhizome. It possesses antioxidant, anti-inflammatory, anti-cancer, anti-arthritis, anti-asthmatic, anti-microbial, anti-viral and anti-fungal properties. This review aims to provide an overview of the potential health benefits of curcumin to treat female reproductive disorders, including polycystic ovary syndrome (PCOS), ovarian failure and endometriosis. Comprehensive information on curcumin was retrieved from electronic databases, which were MEDLINE via EBSCOhost, Scopus and Google Scholar. The available evidence showed that curcumin reduced the high level of androgen in PCOS. Studies in rodents suggest that curcumin resulted in the disappearance of cysts and the appearance of healthy follicles and corpora lutea. Furthermore, animal studies showed curcumin improved the overall function of the ovary in ovarian diseases and reversed the disturbance in oxidative stress parameters. Meanwhile, in vitro and in vivo studies reported the positive effects of curcumin in alleviating endometriosis through anti-inflammatory, anti-proliferative, anti-angiogenic and pro-apoptotic mechanisms. Thus, curcumin possesses various effects on PCOS, ovarian diseases and endometriosis. Some studies found considerable therapeutic effects, whereas others found no effect. However, none of the investigations found curcumin to be harmful. Curcumin clinical trials in endometriosis and ovarian illness are still scarce; thus, future studies need to be conducted to confirm the safety and efficacy of curcumin before it could be offered as a complementary therapy agent.

## 1. Introduction

Turmeric or *Curcuma longa* is a rhizomatous herbal plant from the Zingiberaceae family. This plant is easily found throughout tropical Asia, including India and South China, Southeast Asia, Papua New Guinea and northern Australia [1]. In Asian countries, turmeric is widely used in food and medicine as a natural colouring and flavouring agent at a concentration range of 5–500 mg/kg [2]. Traditionally, turmeric has been used to treat digestive disorders, rheumatoid arthritis, conjunctivitis, liver ailment, urinary tract infection, smallpox, chickenpox, wounds and regulation of menstruation [3]. Turmeric has more than 300 biologically active components, such as polyphenols, sterols, diterpenes, sesquiterpenes, triterpenoids and alkaloids [3]. Turmeric rhizome contains a high number of polyphenols (bioflavonoids) called curcuminoids, which comprise curcumin (60–70%), demethoxycurcumin (20–30%) and bisdemethoxycurcumin (10–15%) [4]. The yellow colour of turmeric is due to the content of curcuminoids [5]. Considering its high content, curcumin is a well-studied compound. Numerous studies on curcumin have demonstrated its many benefits due to its antioxidant, anti-inflammatory, anti-cancer, anti-arthritis, anti-asthmatic, anti-microbial, anti-viral and anti-fungal properties [6,7,8,9,10,11,12,13].

**Figure 1:**
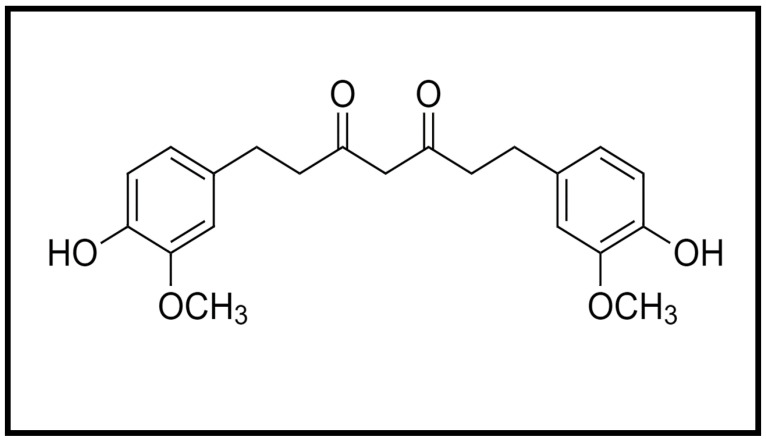
Chemical structure of curcumin.

Curcumin is a hydrophobic molecule known as diferuloylmethane, with the chemical formula C_21_H_20_O_6_ and a molecular weight of 368.38 (Figure 1). Three reactive functional groups have been determined in curcumin: one diketone moiety and two phenolic groups. Among the biological and chemical reactions of curcumin are hydrogen donation reactions leading to oxidation of curcumin, reversible and irreversible nucleophilic addition reactions, hydrolysis, degradation and enzymatic reactions [4].

**Figure 2:**
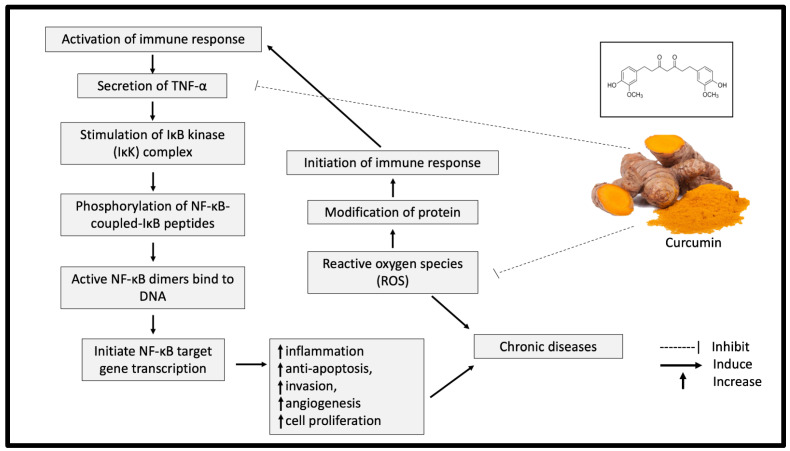
Potential inhibitory effect of curcumin on inflammation and oxidative stress. TNF-α: tumor necrosis factor alpha; NF-κB: nuclear factor kappa B; IKK: IκB kinase.

Curcumin exhibits significant antioxidant properties by breaking the chain reaction of free radical production. The ability of curcumin to capture hydrogen peroxide is also higher compared with the same concentration (20 mM) of commercial antioxidants, such as butylated hydroxyanisole (BHA), butylated hydroxytoluene (BHT) and vitamin E [14]. A meta-analysis study further supported this finding by indicating a significant reduction of oxidative stress markers, including superoxide dismutase (SOD), catalase (CAT), glutathione peroxidase (GSH) and lipid peroxides after curcumin supplementation [15]. In addition, curcumin reduced the protein responses involved in inflammation processes, such as those of tumour necrosis factor alpha (TNF-α), interleukin-1 (IL-1), IL-2, IL-6, IL-8 and IL-12 [16]. TNF-α is a significant mediator of inflammation, which eventually leads to chronic diseases [17]. The presence of TNF-α activates nuclear factor (NF)-κB and amplifies the inflammatory responses [18]. Curcumin has been shown to inhibit the activation of TNF-α in the NF-κB pathway and neutralise the reactive oxygen species (ROS), causing oxidative stress (Figure 2) [18]. As oxidative stress and inflammation are implicated in most chronic diseases, curcumin supplementation could significantly offer various health benefits.

Owing to its therapeutic potentials, various curcumin products have become available in the market in the form of tablets, capsules, drinks, ointments and cosmetics [19]. The broad use of curcumin in India, Japan, Thailand, China, Korea, Malaysia, Pakistan and the United States raises the safety level of curcumin consumption [20]. As a result, the US Food and Drug Administration (US FDA) has labelled curcumin as a “Generally Recognised As Safe” (GRAS) product [19]. Furthermore, the Joint United Nations and World Health Organization Expert Committee on Food Additives (JECFA) and European Food Safety Authority (EFSA) recommended the daily intake of 0–3 mg/kg body weight of curcumin [21]. In addition, curcumin supplementation in several clinical trials demonstrated good tolerability and safety profiles at doses between 4000 and 8000 mg/day [22].

**Figure 3:**
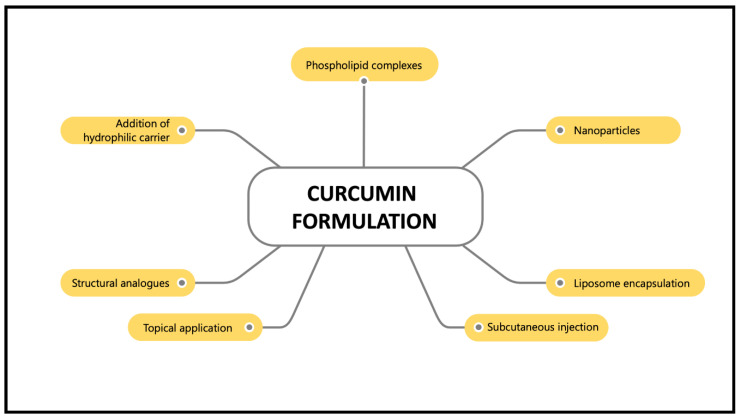
Various curcumin formulations that enhance its bioavailability.

However, the main drawback of curcumin supplementation is its poor bioavailability, which is characterised by its poor absorption, rapid metabolism and rapid elimination [23]. Normally, curcumin is metabolised in the liver and intestines [24]. It is converted into water-soluble metabolites (glucuronides and sulphates) and excreted in the urine [23]. Oral administration of curcumin in rats showed 40% excretion in the faeces [25]. Thus, various curcumin nanoformulations, such as nanoparticles, liposome encapsulation, phospholipid complexes, structural analogues, subcutaneous injection, topical application and hydrophilic carrier addition have been introduced to maximise their bioavailability and activity and preventing curcumin from hydrolysis inactivation (Figure 3). These nanoformulations work by improving curcumin particle size, surface area, surface charge, hydrophobicity, as well as enhancing small intestine permeation [26]. Furthermore, curcumin nanoformulations have been reported to increase absorption more than 100-fold compared to the unformulated curcumin [27]. For example, curcumin has been formulated in a nano biocompatible form (nanocurcumin) to increase its water solubility and bioavailability. Co-administration of curcumin with piperine, an alkaloid in black pepper, enhances curcumin’s bioavailability by up to 2000% [28]. A synthesised nanoparticle form of curcumin, theracurmin, showed satisfactory plasma concentration after a single dose in healthy controls and cancer patients [29]. In addition, the encapsulation of curcumin in liposomes demonstrated inhibitory effects on endometrial cancer by inducing cell apoptosis, inhibiting cell proliferation and inhibiting invasive and metastatic bioactivities [30]. Due to the variety of effective formulations available nowadays, problems regarding the poor bioavailability of curcumin should no longer be the primary concern. Hence, these curcumin nanoformulations have made a significant contribution to the pharmaceutical industry and have been demonstrated to be beneficial in treating various human diseases [26].

Reviews on the therapeutic potentials of curcumin on female reproductive disorders, including polycystic ovary syndrome (PCOS), ovarian failure and endometriosis, remain scarce. Therefore, this review aims to summarise the potential health benefits of curcumin on female reproductive disorders.

## 2. Methods of Review

**Figure 4:**
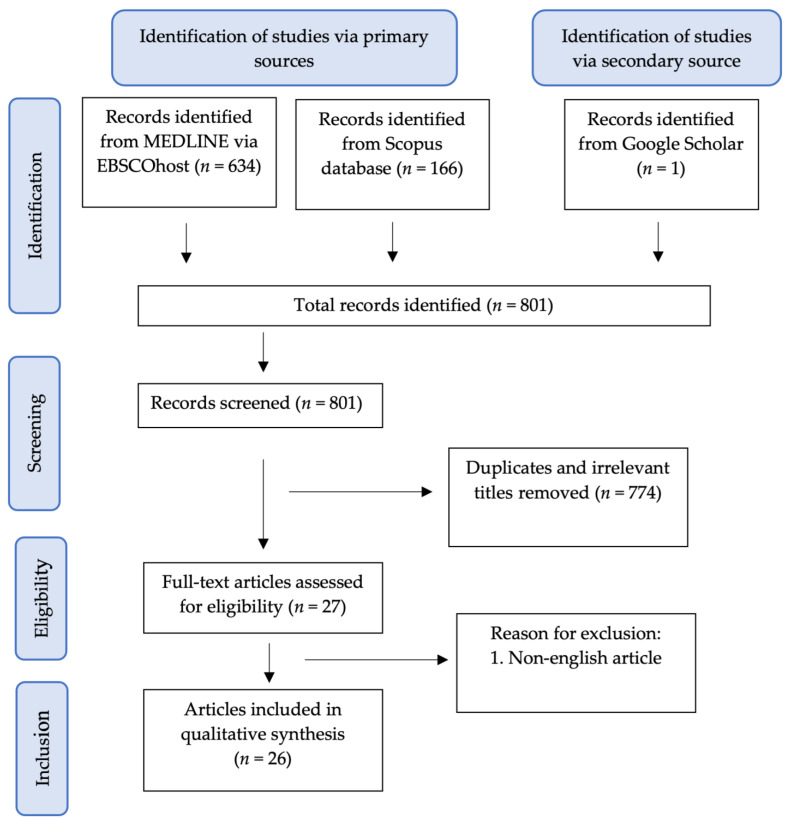
Flowchart of the article selection process.

A literature search was undertaken to identify relevant articles related to the therapeutic potentials of curcumin in female reproductive disorders. Peer-reviewed and full-text English articles published from 2000 to March 2021 were gathered in electronic databases, including MEDLINE via EBSCOhost, Scopus and Google Scholar. The following keywords were used: (1) Curcumin and (2) PCOS or Endometriosis or Ovarian Disease. Only studies involving curcumin treatment on PCOS, endometriosis and ovarian disease were included for this review. The literature search was summarised in the flowchart in Figure 4.

The selection of articles involved two phases. In the first phase, the titles and abstracts were screened, and any articles that did not match the inclusion criteria were excluded. In the second phase, the remaining articles were retrieved and screened thoroughly by all authors. Any differences in opinion were resolved by the discussion between the authors.

The following data were recorded from the studies: the type and age of used samples, the treatment given to the subjects, the type of analysed parameters and the method of analysis, and the results and conclusion of the studies.

## 3. Effect of Curcumin on Polycystic Ovary Syndrome (PCOS)

PCOS is a complex disease; it is a combination of endocrine, reproductive, metabolic and psychological disorders [31]. PCOS affects around 6–20% of women’s reproductive age, depending on the diagnostic criteria [32]. The pathophysiology of PCOS has not been fully clarified, and currently, only symptomatic treatments have been administered to patients with PCOS [33,34]. Numerous herbal remedies have been tested on women with PCOS and PCOS-induced animal models [35,36,37]. These remedies include green tea, welsh onion root and Chinese herbal medicine [38,39,40]. Curcumin has been studied for its potential in PCOS treatment. We found four clinical trials (Table 1) and five in vivo studies (Table 2) that investigated the effects of curcumin on women with PCOS and PCOS-induced animals. All these studies reported different degrees of curcumin benefits to PCOS. However, no adverse effect has been reported.

One of the main features of PCOS is the elevated level of androgens [50]. Curcumin has been shown to reduce the high level of androgen in PCOS in three studies. A randomised controlled clinical trial involving 67 women with PCOS treated with 500 mg curcumin powder for 12 weeks three times daily showed that the level of dehydroepiandrosterone was decreased compared with the placebo group [42]. Meanwhile, two animal studies reported that the elevated testosterone level in PCOS-induced rats was reduced after 100 and 200 mg/kg curcumin and 50 and 100 mg/kg nano curcumin treatments [47,49]. Apart from disrupted androgen levels, PCOS also affected women’s LH/FSH ratio balance [51]. However, a randomised controlled clinical trial showed that curcumin has no effects on LH, FSH and oestrogen levels in PCOS women [42]. According to the researchers, these might be due to the different health statuses of study participants and different doses and treatment durations used in different studies [42]. In animal studies, 50 mg/kg nano curcumin and 200 mg/kg curcumin treatment reversed the reduction in oestradiol and progesterone levels in PCOS-induced rats [47,49]. Curcumin has oestrogenic properties, which might explain its effects on hormone levels [52].

Insulin resistance is one of the pathophysiologies of PCOS [53]. Nearly half of the women diagnosed with PCOS have insulin resistance, which causes hyperinsulinemia [50]. Curcumin has various effects on insulin levels and insulin resistance in women with PCOS. Three clinical trials investigated the effect of curcumin on insulin resistance in women with PCOS. However, only one study proved the benefit of curcumin compared with placebo. In a randomised control trial, women with PCOS were administered with 500 mg/day curcumin for 12 weeks, which reduced the serum insulin, homeostatic model assessment of insulin resistance (HOMA-IR) and fasting glucose levels, as well as increased quantitative insulin sensitivity check index (QUICKI) [41]. Another randomised trial on women with PCOS who were given curcumin at 500 mg three times daily for 12 weeks only showed a reduction in fasting blood glucose, but no effect was found on fasting insulin, HOMA-IR and QUICKI [42]. A randomised study involving overweight or obese women with PCOS reported that administration with 500 mg curcumin twice daily decreased serum insulin level and increased QUICKI after curcumin administration for six weeks. However, comparing the curcumin-treated and placebo groups, no difference was found in serum insulin and QUICKI [43]. The inconsistent findings of curcumin effects on insulin and glucose levels in PCOS women might be due to the participants’ varying ages, health statuses and different inclusion criteria. For example, one study included impaired glucose tolerance as an inclusion criterion [42], while another excluded diabetic participants [43]. Hence, more studies should be conducted to clarify the discrepancy in curcumin’s effect on insulin resistance in PCOS. Meanwhile, a study on PCOS-induced rats reported that treatment with 50, 100 and 200 mg/kg nano curcumin increased the expression of pancreatic PI3K/AKT/mTOR protein levels, which are the pathways reportedly associated with defective insulin level and insulin resistance [47,54]. This finding might explain the possible mechanism of curcumin on altering the insulin pathway.

Obesity is a risk factor for the development of PCOS [55,56]. It is estimated that 38–88% of women with PCOS are overweight or obese [43]. A modest weight loss of 5–10% results in clinically meaningful improvements in PCOS features [55]. In two clinical trials, Jamilian et al. [41] found a significant reduction in body weight and BMI in curcumin-treated women with PCOS, but Heshmati et al. [42] found no changes in BMI and waist circumference. In both trials, women with PCOS of almost the same range of BMI were involved; the trials lasted for 12 weeks, and the same amount of curcumin was used (500 mg). However, Heshmati administered curcumin three times a day, whereas Jamilian et al. only administered curcumin once a day. Thus, a disparity exists in the weight-loss potential, implying that more research is needed.

Curcumin has been tested for its lipid profile improvement properties in animals and humans. In a clinical trial, 500 mg/day curcumin reduced total cholesterol, low-density lipoprotein (LDL)-cholesterol and total-/HDL cholesterol ratio and increased high-density lipoprotein (HDL)-cholesterol levels in women with PCOS [52]. However, another clinical trial did not find any difference in cholesterol, triglyceride, LDL and HDL levels after curcumin treatment in women with PCOS [43]. In two studies that used PCOS-induced rats, 50 mg/kg of nano curcumin and 100 mg/kg of curcumin improved triglyceride, total cholesterol and LDL and HDL cholesterol levels [47,49]. Further studies into curcumin mechanism of action in lowering lipid might explain the discrepancies that exist.

The therapeutic potentials of curcumin are attributed to its anti-inflammatory properties [57]. In two animal studies, 100, 200, 300 and 400 mg/kg curcumin treatment on PCOS-induced rats reduced interleukin-6 and C-reactive protein [45,48]. Nanocurcumin treatment also decreased TNF-α levels in PCOS-induced rats [44]. However, in a clinical trial on women with PCOS, curcumin treatment did not affect high-sensitivity C-reactive protein (hs-CRP) [43].

PCOS is the leading cause of anovulatory infertility [31]. Curcumin has the potential of improving ovarian function in PCOS, as shown in animal studies. In a study by Abuelezz et al., treatment with nano curcumin showed thickened granulosa cells and oocyte appearance in PCOS-induced rats [47]. Another study reported a significant reduction in thickness of theca layer and increased corpus luteum diameter in the curcumin-treated group compared with the PCOS group [48]. Moreover, curcumin treatment displayed results comparable with those of clomiphene citrate, the first-line therapy for anovulatory PCOS, which resulted in the disappearance of cysts and the appearance of healthy follicles and corpora lutea [49]. However, another study found that 5.4 mg/100 g curcumin in nanoparticle form reduced the total number of primary, secondary, antral and primordial follicles compared with the PCOS rat group [46]. These contradictory discoveries of curcumin’s effects on ovarian follicles are expected due to different methods used in inducing PCOS animal models as well as the different forms of curcumin used.

Oxidative stress is involved in the pathophysiology of PCOS [58]. A systematic review and meta-analysis of oxidative stress markers involving 4933 women with PCOS and 3671 controls found homocysteine, malondialdehyde (MDA), dimethylarginine, SOD, glutathione and araoxonase-1 levels were abnormal in women with PCOS [59]. Curcumin possesses excellent antioxidant potential [60,61]. A randomised controlled clinical trial on 72 women with PCOS reported that supplementation 1500 mg curcumin three times daily for three months resulted in the increased activity of the GSH enzyme and peroxisome proliferator-activated receptor γ coactivator 1α [44]. However, the same study did not find any difference in gene expression of sirtuin-1 and the activity of the SOD enzyme [44]. In animal studies, nano curcumin at 50, 100 and 200 mg/kg doses decreased the MDA level and increased GSH and SOD activities in PCOS-induced rats [47]. Another study in PCOS induced rats showed that 100 and 200 mg/kg curcumin increased SOD and CAT activities, whereas the TBARS level decreased and the GSH level increased only at 200 mg/kg curcumin [49].

Curcumin has been shown to inhibit apoptosis in some diseases while promoting it in others [62,63,64]. Nanoparticle curcumin treatment reportedly decreased Bcl-2-associated X protein (BAX) and caspase3 (CASP3) protein expression and increased the levels of B-cell lymphoma 2 (Bcl2) expression. This study also showed reduced apoptosis in the granulosa cells of PCOS-induced rats after curcumin treatment [46].

Many discrepancies remain in the effect of curcumin on PCOS, especially in clinical trials. However, the animal and human studies discussed above have provided an excellent foundation for curcumin’s potentials as PCOS complementary treatment; thus, further investigation is warranted to endorse the use of curcumin in the future.

## 4. Effect of Curcumin on Ovarian Diseases

Curcumin has beneficial effects on ovary-related diseases that could be achieved through its anti-inflammation, anti-apoptosis and antioxidant properties. Table 3 outlines the effects of curcumin on ovarian disorders. In one study, mice with D-galactose induced premature ovarian failure were treated with 100 mg/kg/day curcumin intraperitoneally for 42 days [65]. In this study, curcumin has been shown to inhibit d-galactose-induced oxidative stress, apoptosis and ovarian injury. Increased SOD and decreased MDA levels, as well as reduced SOD2 and CAT mRNA expression levels, were found in the curcumin-treated group. In addition, this study also showed that curcumin increases NF-E2-related factor-2 (Nrf2) and HO-1 protein expression levels, which are the proteins involved in the mechanism of ROS removal [65]. Nrf2 can bind to antioxidant response elements (AREs) in the promoter region of Nrf2 target genes, which used HO-1 sequential enzymatic processes to eliminate ROS [66].

In another study, a rat model of ovarian ischemia–reperfusion injury was administered with 200 mg/kg curcumin intraperitoneally, simultaneously with reperfusion. It was found that curcumin administration did not alter nitric oxide (NO), NO synthase (NOS), xanthine oxidase (XO), total antioxidant status (TAS) and total oxidant status (TOS) [68]. Interestingly, in another rat model of ovarian ischemia–reperfusion injury, 1 mg/kg nano curcumin improved the oxidative status parameters but not 100 mg/kg. These results included significantly higher values of superoxide dismutase, total glutathione, GSH, glutathione reductase and glutathione S-transferase and substantially lower values of nitric oxide synthase, MDA, myeloperoxidase and 8-hydroxy-2 deoxyguanine levels [69]. The findings from these two studies showed that nanocurcumin at very low concentrations (1 mg/kg) produced significant improvements compared to the native curcumin (100 mg/kg and 200 mg/kg curcumin), which might be attributed to its high bioavailability and longer half-life.

In a more specific oxidative stress study, Kunming mice were injected with 8 mg/kg sodium arsenite to induced ovarian oxidative stress and treated with 100, 150 or 200 mg/kg curcumin once per day for 21 days. Curcumin reversed the disturbance in oxidative stress parameters (ROS, MDA and SOD, except for GSH) induced by sodium arsenite. Furthermore, in this study, p66Shc expression, upregulated under oxidative stress, was significantly lowered by curcumin treatment [71]. The p66Shc protein reportedly involves signalling pathways that regulate the cellular response to oxidative stress and cell lifespan. Thus, when the expression of p66Shc is upregulated, the generation of ROS increases and the oxidative damage to cells becomes severe [72].

Curcumin improves the overall function of the ovary in ovarian diseases. Curcumin administration at 100 mg/kg/day to premature ovarian failure mice increased the number of primordial follicles and increased AMH expression levels, which reflected the size of the ovarian follicle pool [65,73]. Meanwhile, administration of curcumin at 100, 150 and 200 mg/kg once per day reduced the sodium arsenite-induced increment in the atretic follicle [71]. In the mice model of autoimmune disease of the ovaries, 100 μg/g curcumin treatment four times a week alleviated the reduced level of oocytes in metaphases I and II [70].

The anti-apoptotic effect of curcumin on the ovary has been reported in several studies. Curcumin (100 mg/kg/day) administration reduced apoptosis of granulosa cells and reduced cleaved caspase-3 and -9 protein expression levels in mice with D-galactose-induced premature ovarian failure [65]. An in vitro study on porcine ovarian granulosa cells demonstrated that curcumin reportedly reduced PCNA and its mRNA, as well as increased both Bax and its mRNA and reduced cell viability [67]. However, in the mice model of autoimmune ovary disease, 100 μg/g curcumin did not alter the number of apoptotic cells in the thymus, spleen and lymph node but reduced the necrosis in these cells [70].

## 5. Effect of Curcumin on Endometriosis

Endometriosis is a chronic gynaecological disorder representing the implantation of endometrial glands and stroma outside the uterine cavity [74]. It affects adolescents and reproductive-aged women and is commonly associated with infertility, dyspareunia, dysmenorrhea and chronic pelvic pain [75]. The pathogenesis of endometriosis has not been fully understood. To date, nearly all current treatment options for endometriosis suppress endometrial function and are not curative. Combined oral contraceptives and progestins are commonly prescribed as first-line therapy to alleviate pain symptoms. However, if the first-line therapies are ineffective, contraindicated or not tolerated, gonadotropin-releasing hormone-agonists are prescribed. In case of resistance to other treatments, an aromatase inhibitor is prescribed [76]. Presently, curcumin was found to have anti-endometriosis, antioxidant and anti-inflammatory properties [77].

**Figure 5:**
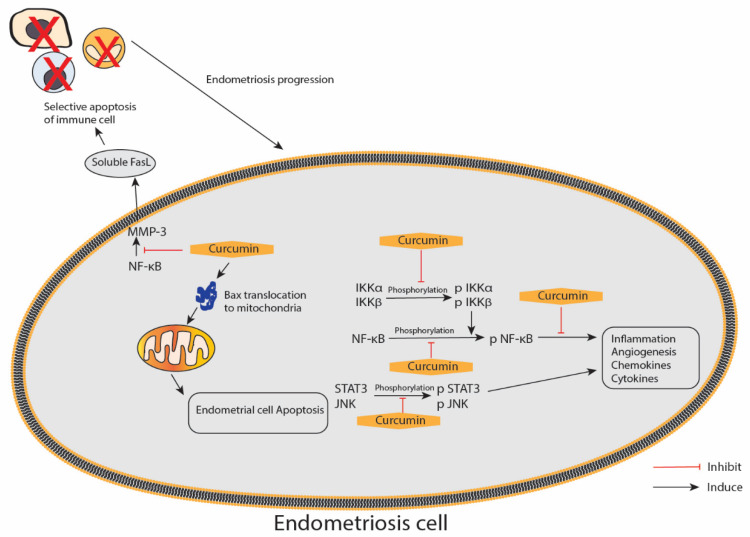
An illustration of the proposed curcumin effect on the inflammatory and apoptotic pathways in endometriosis. IKKα/β—inhibitor of nuclear factor κ-B kinase subunit α/β; JNK—c-Jun N-terminal kinases; NF-κB—nuclear factor κ-light-chain-enhancer of activated B; STAT3—signal transducer and activator of transcription 3; p—phosphorylated form; MMP-3—matrix metalloproteinase 3; FasL—Fas ligands; arrow represents a promotion, and blunt arrow represents inhibition.

In this review, we found six studies that utilised the animal model of endometriosis, which involved the transplantation of endometrium derived from the animal endometrium, and five in vitro studies that explored the effect of curcumin against endometriosis (Table 4: animal (in vivo) studies; Table 5: in vitro studies). All these studies reported different degrees of curcumin’s advantage on endometriosis in terms of therapeutic potential. In addition, the studies reported the positive effects of curcumin on the endometrium, particularly those exerted through anti-inflammatory, anti-proliferative, anti-angiogenic and pro-apoptotic mechanisms. Figure 5 illustrates the proposed effect of curcumin on the inflammatory and apoptotic pathways in endometriosis.

The anti-inflammatory effects of curcumin are mediated through interference with the expression or activation of multiple key signalling molecules, including nuclear factor-κB [87]. In a study conducted by Kim et al., curcumin effectively suppressed ICAM-1 and VCAM-1 gene and protein expressions and the secretions of IL-6, IL-8 and MCP-1 by inhibiting the activation of NF-κB induced by TNF-α, which is a central proinflammatory cytokine in the endometriotic disease process, in human ectopic human endometriotic stromal cells [82]. Furthermore, CAM-1 and VCAM-1 protein expressions in TNF-α-activated endometriotic stromal cells after curcumin treatment measured by immunofluorescence microscopic and Western blot analyses were in accordance with the ICAM-1 and VCAM-1 expression patterns, as shown by qRT-PCR analysis, thereby indicating that both cell surface and cytoplasmic proteins, as well as the mRNA expressions of ICAM-1 and VCAM-1 in endometriotic stromal cells induced by TNF-α, were inhibited by curcumin [82].

Eutopic endometrial cells function differently in women with endometriosis compared with disease-free women with normal endometrium. The cells are resistant to apoptosis and have other selective advantages for survival outside the uterine cavity, leading to their implantation and invasion of the peritoneum and other ectopic sites [88]. A study conducted by Chowdhury et al. reported increased chemokines and cytokines produced in eutopic endometrial tissue from women with endometriosis might increase angiogenesis and proliferation. The study used a primary cell culture of human endometriotic stromal cells from women with endometriosis (EESCs) and normal endometrial stromal cells (NESCs) as subjects, which were then treated with curcumin at different doses of 1, 5, 10, 20 and 40 μg/mL for 24, 48 and 72 h. The various secretions of chemokines and cytokines, i.e., IL-6, IL-8, IP-10, G-CSF, MCP-1 and RANTES, were highly expressed in EESCs. However, IL-10 and IL-12 expression levels did not differ between EESCs and NESCs. After the treatment with curcumin, a significant inhibition towards the secretion of IL-6, IL-8, IP-10, G-CSF, MCP-1 and RANTES in EESCs after 48 h was observed. In contrast, curcumin significantly promoted IL-10 and IL-12 secretions in EESCs after 48 h. IL-17 was utterly absent in the media after treatment with curcumin for 24 h. The Western blot result showed that curcumin inhibited the phosphorylation of IKKα, IKKβ and NF-κB in EESCs. Moreover, it significantly inhibited the phosphorylation of JNK and STAT3 in EESCs. JNK expression also significantly decreased after curcumin treatment. Hence, curcumin has therapeutic potential and nullifies the abnormal activation of chemokines and cytokines, IKKα/β, NF-κB, STAT3 and JNK signalling pathways to reduce inflammation, which is associated with endometriosis [83].

Angiogenesis is essential in the pathogenesis of endometriosis, as it establishes a new blood supply critical for developing endometriotic lesions [89]. VEGF is a signalling protein produced by cells; it stimulates angiogenesis, vasculogenesis and vascular permeability [90]. McLaren et al. demonstrated that the VEGF levels increased in the peritoneal fluid of patients with endometriosis [91]. Angiogenesis is essential in developing endometriosis and is regulated by a variety of pro-angiogenic genes and signalling molecules, including VEGF. Curcumin exhibited an anti-angiogenic effect in two studies, namely, one animal study and one in vitro study. Hong Cao et al. reported that after the treatment with 20 and 50 μmol/L curcumin in vitro, the proportion of VEGF positive expression decreased compared with the 0 μmol/L groups, and accordingly, the fluorescence intensities of VEGF staining decreased [86]. Another study reported that eutopic and ectopic endometrium expression of VEGF protein in the model rat group was higher than that in the normal group, according to the results of Western blot analysis. VEGF protein expression in the ectopic endometrium of the EMS rats was also higher than in the eutopic endometrium. After curcumin treatment, VEGF protein expression of the ectopic endometrium decreased with increasing doses of curcumin (50, 100 and 150 mg) [79].

Apoptosis is the process of programmed cell death that occurs in multicellular organisms [92,93]. Curcumin may be used in cancer treatment due to its ability to induce apoptosis and inhibit angiogenesis [66,94]. However, the role of curcumin in human endometriotic and endometrial stromal cells remains unclear. Therefore, the study by Cao et al. aimed to determine the apoptotic potential of curcumin in human endometriosis. Treatment with 50 μmol/l curcumin resulted in 4.7% early apoptosis and 28.4% late apoptosis in endometriotic stromal cells, as well as 2.8% early apoptosis and 21.4% late apoptosis in endometrial stromal cells. The data showed that curcumin reduced cell survival in human endometriotic and endometrial stromal cells in vitro [86]. A study by Jana et al. reported that curcumin treatment at 12, 24 and 48mg/kg/daily for three days induced Cyt-c release and caspase-9 expression, suggesting the involvement of mitochondrial pathway in stimulating apoptosis [72]. Curcumin also increased the size of mitochondria, decreased the expression of Cyt-c and increased the expression of Bax within the mitochondria. They also found that curcumin regresses endometriosis via mitochondria-mediated apoptosis by p53-dependent and -independent pathways [74]. Hence, curcumin has been shown to exhibit the pro-apoptotic property in endometriosis.

Although the exact mechanism underlying the development of endometriosis remains unclear, evidence suggests the crucial role of oestrogen in establishing and regulating this disease [95]. Oestradiol (E2) is an essential promoter of the growth of both eutopic and ectopic endometrium. The primary source of E2 is the ovary, and E2 was recently found to be an effective regulator of endometriosis. An in vitro study conducted by Ying Zhang et al. utilised primary cell culture of endometriotic stromal cells, normal endometrial stromal cells, endometriotic epithelial cells and normal endometrial epithelial cells; results revealed that the E2 value of the endometriotic epithelial cells was higher than that of the endometriotic stromal cells. However, the expression of E2 in normal endometrial stromal and epithelial cells was extremely low. WST-8 result showed that ectopic endometriotic stromal cells had a higher growth rate than endometrial stromal cells. After curcumin treatment (10, 30 and 50 μM), the number of endometriotic stromal cells was reduced, and cell growth slowed compared with the 0 μmol/L groups. E2 level was lower after treatment with curcumin, especially in the 30 and 50 μmol/L groups compared with the 0 μmol/L groups. This study showed that curcumin could inhibit the proliferation of endometrial cells by reducing the E2 value [85].

Thus, the available evidence showed that curcumin exerts a beneficial effect against endometriosis. However, further studies are required to fully elucidate the molecular mechanisms underlying the effects of curcumin and investigate its potential to treat endometriosis in humans.

## 6. Conclusions

Curcumin has many different effects on PCOS, ovarian diseases and endometriosis. Some studies found considerable therapeutic effects, whereas others found no effect. However, none of the investigations found curcumin to be harmful. Curcumin has been evaluated in clinical trials involving women with PCOS. The results of these trials may serve as the foundation for further exploration of the curcumin potential as a complementary therapy to the existing treatment plan. Additionally, there is a lack of human clinical trials to validate the effects of curcumin on endometriosis and ovarian diseases; hence, it warrants further investigation. Meanwhile, research on curcumin has grown extensively with various curcumin supplements that have become available in the market in the form of tablets, capsules and drinks. However, the use of curcumin supplements in the prevention and complementary treatment of female reproductive diseases is still limited. Thus, future studies should be conducted to assess its efficacy in managing female reproductive diseases and prove the safety of these supplements.

## Figures and Tables

**Table 1:** Summary of the effects of curcumin on PCOS in humans.

Type of Model	Treatment and Treatment Duration	Findings	References
A randomised, double-blind, placebo-controlled trial on 60 women with PCOS aged from 18 to 40 years old	1. 500 mg/day curcumin 2. Placebo Orally 12 weeks	Curcumin significantly reduced the following parameters compared with the placebo: 1. weight and BMI; 2. fasting glucose; 3. serum insulin; 4. HOMA-IR (insulin resistance); 5. total cholesterol; 6. LDL-cholesterol; 7. total-/HDL cholesterol ratio Curcumin significantly increased the following parameter compared with the placebo: 1. HDL-cholesterol levels; 2. QUICKI (insulin sensitivity); 3. gene expression of peroxisome proliferator-activated receptor-gamma (PPAR-γ); 4. gene expression of low-density lipoprotein receptor (LDLR)	[41]
A randomised, double-blind, placebo-controlled clinical trial on 67 women with PCOS aged from 18 to 49 years old	1. 500 mg curcumin powder in a capsule 2. Placebo (maltodextrin) capsules Orally 3 times daily for 12 weeks	-Decreased fasting plasma glucose (FPG) and dehydroepiandrosterone levels in the curcumin-treated group compared with the placebo -Statistically non-significant increase in oestradiol levels -No changes in fasting insulin, LH and FSH, homeostatic model assessment of insulin resistance (HOMA-IR), quantitative insulin sensitivity check index (QUICKI), BMI and waist circumference among different groups	[42]
A randomised, double-blind, placebo-controlled clinical trial on 60 overweight or obese women with PCOS	1. 500 mg curcumin twice daily 2. Placebo Orally 6 weeks	-Intragroup analysis of serum insulin decreased, whereas QUICKI increases significantly in the curcumin-treated group -No significant changes were recorded in all parameters between the curcumin-treated and placebo groups, including FBS, insulin, HOMA-IR, QUICKI, total cholesterol, triglyceride, LDL and HDL and high-sensitivity C-reactive protein (hs-CRP) levels	[43]
A randomised, double-blind, placebo-controlled clinical trial on 72 women with PCOS	1. 1500 mg curcumin 3 times daily 2. Placebo Orally 3 months	Significantly increased gene expression of peroxisome proliferator-activated receptor γ coactivator 1α and the glutathione peroxidase enzyme activity -Non-significantly increased gene expression of sirtuin-1 and activity of the superoxide dismutase (SOD) enzyme	[44]

Abbreviations: BMI: body mass index; HOMA-IR: homeostatic model assessment of insulin resistance; QUICKI: quantitative insulin sensitivity check index; LDL cholesterol: low-density lipoprotein cholesterol; HDL cholesterol: high-density lipoprotein cholesterol; LH: luteinising hormone; FSH: follicle-stimulating hormone; PPAR-γ: peroxisome proliferator-activated receptor-gamma; LDLR: low-density lipoprotein receptor; FPG: fasting plasma glucose; hs-CRP: high-sensitivity C-reactive protein; SOD: superoxide dismutase.

**Table 2:** Summary of the effects of curcumin on PCOS in animals.

Type of Model	Treatment and Treatment Duration	Findings	References
Adult female Wistar rats treated with oestradiol valerate to induce PCOS	1. 100 mg/kg curcumin 2. 300 mg/kg curcumin Method of administration not specified 14 days	-Reduced number of insulin resistance index (HOMA-IR decreased, QUICKI increased) -Reduced interleukin-6 and C-reactive protein -Reduced necrotic liver cells	[45]
Prepuberal BALB/c female mice treated with DHEA to induce PCOS	5.4 mg/100 g curcumin in the form of curcumin-loaded super-paramagnetic iron oxide (Fe_3_O_4_) nanoparticles Intraperitoneally 20 days	-Reduced ovarian volume and total number of primary, secondary, antral and primordial follicles compared to the PCOS and vehicle groups -Significantly decreased Bcl-2-associated X protein (BAX) and levels of expression of Caspase3 (CASP3) protein; increased levels of B-cell lymphoma 2 (Bcl2) expression and moderated apoptosis in granulosa cells compared with PCOS group	[46]
Adult female Wistar rats treated with letrozole to induce PCOS	1. Nanocurcumin (50, 100 and 200 mg/kg) 2. Clomiphene citrate (1 mg/kg) Orally 15 days	-Nanocurcumin (50 mg/kg) and clomiphene citrate attenuated the PCOS-induced reduction in oestradiol and progesterone levels -Nanocurcumin (50 and 100 mg/kg) and clomiphene citrate attenuated the PCOS-induced testosterone increment -Nanocurcumin (50 mg/kg) and clomiphene citrate improved triglyceride, total cholesterol, and LDL and HDL cholesterol levels -All doses of curcumin and clomiphene citrate reduced the PCOS-induced increment of fasting blood glucose and insulin -Clomiphene citrate and curcumin alleviated insulin resistance -Clomiphene citrate and curcumin decreased the MDA level and increase GSH and SOD activity -Clomiphene citrate and curcumin decreased TNF-α levels -Clomiphene citrate and curcumin increased the protein expression of PI3K/AKT/mTOR levels -Treatment with nanocurcumin showed thickened granulosa cells and the appearance of oocytes in a dose-dependent manner -Nano curcumin treatment and clomiphene retained the pancreatic tissue integrity and caused a gradual increase in the area of the islet and count of β-cells	[47]
Adult female Wistar rats treated with oestradiol valerate to induce PCOS	Curcumin (100, 200, 300 and 400 mg/kg) Intraperitoneally 14 days	-Significant reduction in thickness of theca layer and increased corpus luteum diameter in the curcumin-treated group compared with the PCOS group -Curcumin decreased the IL-6 and CRP levels -Curcumin decreased TNF-α in the granulosa layer and follicular fluid	[48]
Adult female Wistar rats treated with letrozole to induce PCOS	1. Curcumin (100 and 200 mg/kg) 2. 1 mg/kg clomiphene citrate Orally 15 days	-Curcumin significantly inhibited the decrease in uterine weight -Clomiphene citrate and 100 and 200 mg/kg curcumin reversed the disturbance in testosterone and progesterone levels in PCOS-induced rats, whereas only clomiphene and 200 mg/kg curcumin effectively normalised the oestrogen level -Both doses of curcumin reduced fasting blood glucose and HbA1c levels -Clomiphene and 100 and 200 mg/kg curcumin decreased triglyceride, total cholesterol and LDL levels, whereas only 200 mg/kg curcumin increased HDL level -100 and 200 mg/kg curcumin increased SOD and CAT activity, and only 200 mg/kg curcumin decreased the TBARS level and increased GSH level -Clomiphene increased catalase activity, reduced TBARS and showed no effect on GSH and SOD -Clomiphene citrate and both curcumin doses resulted in the disappearance of cysts and the appearance of healthy follicles and corpora lutea	[49]

Abbreviations: HOMA-IR: homeostatic model assessment of insulin resistance; QUICKI: quantitative insulin sensitivity check index; LDL cholesterol: low-density lipoprotein cholesterol; HDL cholesterol: high-density lipoprotein cholesterol; BAX: Bcl-2-associated X protein; CASP3: caspase3; Bcl2: B-cell lymphoma 2; MDA: malondialdehyde; TBARS: thiobarbituric acid reactive substances; GSH: glutathione peroxidase; SOD: superoxide dismutase; CAT: catalase; TNF-α: tumor necrosis factor alpha; PI3K: phosphoinositide 3-kinase; AKT: protein kinase B; mTOR: mammalian target of rapamycin; IL-6: interleukin-6; CRP: C-reactive protein; HbA1c: hemoglobin A1C.

**Table 3:** Summary of the effects of curcumin on ovarian diseases.

Type of Model	Treatment and Treatment Duration	Findings	References
D-galactose-induced premature ovarian failure (POF) in mice	100 mg/kg/day curcumin Intraperitoneally 42 days	-Increased progesterone and oestrogen levels while decreasing FSH and LH levels -Increased SOD and decreased the MDA and SOD2 levels and CAT mRNA expression -Increased primordial follicles -Decreased 8-OhdG, 4-HNE, NTY and senescence-associated protein P16 expression levels -Increased AMH expression levels -Reduced apoptosis in granulosa cells -Increased p-Akt, Nrf2 and HO-1 protein expression levels -Reduced cleaved caspase-3 and -9 protein expression levels	[65]
Porcine ovarian granulosa cells	Curcumin medium at 0, 1, 10 and 100 μg/mL 2 days	-Reduced PCNA and its mRNA, increased Bax and its mRNA, reduced cell viability and stimulated progesterone and testosterone release	[67]
Rat model of ovarian ischemia–reperfusion injury	Curcumin at 200 mg/kg Administered intraperitoneally with reperfusion Group 1: 2 h ischemia and 2 h reperfusion Group 2: 4 h ischemia and 4 h reperfusion Subgroup: (1) Sham: abdominal incision with no ischemia/perfusion (2) Control: abdominal incision with ischemia/perfusion (3) Curcumin: abdominal incision with ischemia/perfusion and curcumin at 200 mg/kg	Group 1: -No significant differences were observed between nitric oxide (NO), NO synthase (NOS), xanthine oxidase (XO), total antioxidant status (TAS) and total oxidant status (TOS) -Significantly higher ovary histological grade in the control and curcumin subgroups compared with the sham subgroup Group 2: -Significantly higher TOS and TAS in the control group than in the sham and curcumin groups -Significantly higher histological grade in the control and curcumin subgroups compared with the sham subgroup -No change in NO, NOS or XO levels	[68]
Rat model of ovarian ischemia–reperfusion injury	1. 100 mg/kg curcumin 2. 1 mg/kg nano curcumin Intraperitoneal Group SSG: laparotomy only Group I: 3 h ischemia only Group I/R: 3 h ischemia and 3 h reperfusion Group I/C: 3 h ischemia only and 100 mg/kg curcumin Group I/R/C: 3 h ischemia, 3 h reperfusion and 100 mg/kg curcumin Group I/NC: 3 h ischemia only and 1 mg/kg nano curcumin Group I/R/NC: 3 h ischemia, 3 h reperfusion and 1 mg/kg nano curcumin	-Nanocurcumin-treated animals showed significantly improved development of ischemia and reperfusion tissue injury compared with other groups -I/R/NC group showed significantly higher superoxide dismutase values, total glutathione, glutathione peroxidase, glutathione reductase and glutathione S-transferase than other groups (*p* < 0.05) -I/R/NC group showed a significantly lower value of nitric oxide synthase, malondialdehyde, myeloperoxidase and 8-hydroxy-2 deoxyguanine levels compared with other Groups -No difference in all biochemical parameters in other groups	[69]
CBA female mice immunised with an extract from the ovaries of outbred albino mice to induce immune disease of the ovaries (model of autoimmune disease in women)	Curcumin at 100 μg/g (four times a week) Intragastric	-The percentage of oocytes in metaphases I and II: Immunised mice: the numbers of oocytes with dissolved germinal vesicle (metaphase I) and the formed polar body (metaphase II) decreased significantly Curcumin-treated mice: the level of oocytes in metaphases I and II increased significantly compared with the immunised group -The number of apoptotic and necrotic cells in the thymus, spleen and lymph node: No changes were recorded in apoptosis cell levels in all groups Immunised mice: significantly increased necrotic cell number in thymus, spleen and lymph node Curcumin-treated mice: Decreased necrotic cell number in thymus, spleen and lymph node compared with the immunised group -Percentage of blood stab neutrophils: Immunised group—increased Curcumin-treated group—decreased -Correlation between parameters of death of immunocompetent cells and number of oocytes resuming meiosis: Significantly negative correlation in immunocompetent cell necrosis and percentage of oocytes in metaphase I for spleen and lymph nodes only	[70]
Female Kunming mice injected with 8 mg/kg sodium arsenite to induce ovarian oxidative stress	0, 100, 150 or 200 mg/kg curcumin once a day Intragastric 21 days	-All doses of curcumin reduced the sodium arsenite-induced increment in ROS value -All doses of curcumin reduced the sodium arsenite-induced increment in MDA value -All doses of curcumin increased the sodium arsenite-induced decrease in SOD value -Decreased sodium arsenite treatment; no change in comparison with curcumin treatment -All doses of curcumin reduced the sodium arsenite-induced increment in the number of atretic follicles. No changes were recorded in primordial, primary and secondary follicles -Curcumin prevented the inhibition of proliferation of granular cells in the sodium arsenite group -p66Shc expression upregulated under oxidative stress was lowered by curcumin	[71]

Abbreviations: LH: luteinising hormone; FSH: follicle-stimulating hormone; 8-OhdG: 8-hydroxy-2’ -deoxyguanosine; 4-HNE: 4-Hydroxynonenal; NTY: nitrotyrosine; AMH: anti-müllerian hormone; Nrf2: nuclear factor erythroid 2–related factor 2; HO-1: heme oxygenase-1; PCNA: proliferating cell nuclear antigen; Bax: Bcl-2-associated X protein; CASP3: caspase3; MDA: malondialdehyde; TBARS: thiobarbituric acid reactive substances; GSH: glutathione peroxidase; SOD: superoxide dismutase; CAT: catalase; TNF-α: tumor necrosis factor alpha; p-Akt: phosphorylated protein kinase B; NO: nitric oxide; NOS: nitric oxide synthase; XO: xanthine oxidase; TAS: total antioxidant status; TOS: total oxidant status; ROS: reactive oxygen species.

**Table 4:** Summary of the effects of curcumin on endometriosis in animals.

Type of Model	Treatment and Treatment Duration	Findings	References
Hetero-transplantation of endometrium tissue of ovariectomised donor mice into the peritoneal cavity containing subcutaneous implant of oestradiol-17β of recipient BALB/c mice	Model: 12, 24 and 48 mg/kg/day via intraperitoneal injection Therapeutic: 48 mg/kg/day curcumin 5 mg/kg, twice/day celecoxib Intraperitoneal injection Mice model: Administered once daily for 1 day prior to the inoculation of the endometrium and continued for another 3 days Therapeutic model (no pre-treatment given prior to inoculation): Curcumin once daily and celecoxib twice daily for the next 5 days	-Pre-treatment with curcumin showed obliteration of glandular regions with sparse infiltration inflammatory cells in the intestinal mucosal layer Curcumin treatment: -Downregulated MMP-3 activity during early regression of endometriosis in a dose-dependent manner -Decreased activity of MMP-3 and increased IκB-α expression leads to reduced NF-κB translocation within the nucleus -Increased in TUNEL positive cells in pre-treated endometriotic tissues as compared with untreated tissue -Induced Cyt-c release and caspase-9 expression suggested the involvement of mitochondrial pathway in stimulating apoptosis -Increased the size of mitochondria, decreased the expression of Cyt-c and increased the expression of Bax within the mitochondria -Distinct lesion of endometriosis was significantly reduced in number and volume by curcumin and moderately by celecoxib -P53 protein expression was upregulated during curcumin treatment but not during celecoxib treatment -Both curcumin and celecoxib activated phosphorylated p38 MAP kinase -The apoptotic response was triggered by increased caspase-9 expression by curcumin and celecoxib compared with normal samples	[74]
Autotransplantation of endometrium tissue of the left horn of the uterus to the abdominal wall and intestinal mesentery of Sprague-Dawley rats	48 mg/kg/day of curcumin 7.2 mg/kg/day of danazol 0.3 mL of ethanol 50% (vehicle) Intraperitoneal injection 4 weeks	-Serum level of leptin in the curcumin treatment group was significantly higher than in all the study groups except the danazol-treated group -No significant difference in serum levels of resistin, homocysteine and CA 125 in the danazol and curcumin treatment groups -Significantly lower serum level TAC in the curcumin-treated group compared with control and danazol-treated group	[77]
Endometriosis with varying severity developed in mice by peritoneal implantation of uterine fragments	(1) 16, 32 and 48 mg/kg/day curcumin (2) 48 mg/kg/day curcumin for therapeutic model (3) PBS (vehicle) Intraperitoneal injection Daily for 3 days Therapeutic model: -Daily for 10 days -Daily for 20 days from day 15 post-endometriosis	At doses of 16, 32 and 48 mg/kg: -Curcumin decreased the secreted matrix metalloproteinase (MMP)-9 activity by 50%, 70% and 80% and the synthesis of MMP-9 decrease significantly by 60%, 70% and 90%, respectively In the therapeutic model: -Curcumin decreased the secreted MMP-9 activity by 45% and 85%, inhibited TNF-α and elevated TIMP-1 -Curcumin decreased lipid peroxidation and protein carbonylation	[78]
Autotransplantation of endometrium tissue during oestrus stage in Wistar rats	50, 100 and 150 mg/kg/d curcumin 0.5% sodium carboxymethyl cellulose solution (vehicle) Intragastric injection Daily for 4 weeks	-Hyperplasia in ectopic tissue was halted -Disappearance of the ectopic gland tissue with the narrowed lumen and sparse cells -Microvessel density (MVD) was higher in ectopic endometrium -In eutopic endometrium, all treated groups showed no significant difference compared with the normal group -Eutopic and ectopic endometrium expression of VEGF protein in the model rat group was higher than in the normal group	[79]
Dimethyl sulfoxide (DMSO) autotransplantation of endometrium tissue from the right horn of the uterus and placed be-tween the peritoneum and muscle of ovariectomised albino Wistar rats	DMSO as vehicle 100 mg/kg of curcumin 100 mg/kg of deferoxamine + curcumin Intragastric Group A: Water for injection daily for 3 days and DMSO Group B: Curcumin Group C: Deferoxamine at 6 h interval for 3 days and curcumin Daily for 20 days	-Endometriotic-like implant sizes were significantly reduced in groups B and C compared with group A (control) -Stroma and glands with surrounding fibrous connective tissue were present and well vascularised in the ectopic endometrium -Cytokeratin-7 antibodies displayed an immunoreactivity in glandular epithelial cells of both eutopic and ectopic endometrium -Blood iron levels were not significantly different between the treatment groups according to atomic absorption spectrophotometry results	[80]
Hetero-transplant of the endometrium of the uterine horn of donor mice into the peritoneal cavity containing subcutaneous implant of oestradiol-17β pellet of recipient BALB/c mice	48 mg/kg/day of curcumin Intraperitoneal injection Daily for 3 days	-No changes were seen in MMP-2 activity for the first 24 h -Both synthesised and secreted proMMP-2 activity were increased after 48 h -Curcumin suppressed the pro-MMP-2 activity, and progression of endometriosis was delayed -Activation of pro-MMP-2 was inhibited; hence, MMP-2 activity was halted by curcumin during the early stage of endometriosis -Upregulation of TIMP-2 inhibited MMP-2 activity during regression of endometriosis by curcumin -MT1MMP regulated MMP-2 activity, as confirmed by immunoblotting. Curcumin downregulated MT1MMP expression by 40% compared with the normal sample -Complex formation between MMP-2 and TIMP-2 was significantly increased in the curcumin-treated group, which inhibited MMP-2 activity and endometriosis progression	[81]

Abbreviations: TNF-α: tumor necrosis factor α; MMP: matrix metalloproteinase; TIMP: tissue inhibitor matrix metalloproteinase; NF-κB: nuclear factor κ-light-chain-enhancer of activated B; MVD: microvessel density; DMSO: dimethyl sulfoxide; VEGF: vascular endothelial growth factor; Cyt-c: cytochrome C; Bax: BCL2 associated X; TAC: total antioxidant capacity; MT1MMP: membrane type-1 matrix metalloproteinase; MAPK: mitogen-activated protein kinase.

**Table 5:** Summary of the effects of curcumin on endometriosis in in vitro studies.

Type of Model	Treatment and Treatment Duration	Findings	References
Cell culture of human endometriotic stromal cells from women with stage III/IV endometriosis	(1) 1, 10, 30 and 50 μM of curcumin (2) 30 μM of curcumin + 15 ng/mL of TNF-α primary (1) 24 h (2) Curcumin for 1 h and stimulated with TNF-α for 15 h for ELISA	-Curcumin did not affect the viability of endometriotic stromal cells at doses up to 50 μM -Pre-treatment with curcumin significantly suppressed TNF-α-induced ICAM-1 and VCAM-1 mRNA expression in a dose-dependent manner -Curcumin significantly suppressed TNF-α-induced ICAM-1 VCAM-1 cell surface expression in a dose-dependent manner -Pre-treatment with curcumin (30 μM) markedly suppressed TNF-α-induced total cellular protein expression of ICAM-1 and VCAM-1 -Curcumin (30 μM) suppressed the TNF-α-induced IL-6, IL-8 and MCP-1 from endometriotic stromal cells -Pre-treatment with curcumin (30 μM) blocked the TNF-α-induced nuclear translocation of NF-κB p65 from the cytosol into the nucleus and strongly inhibited TNF-α-induced phosphorylation and degradation of IκB -Curcumin (30 μM) significantly reduced the density of the NF-κB shifted band that was induced by TNF-α	[82]
Primary cell culture of human endometriotic stromal cells from women with endometriosis (EESCs) and normal endometrial stromal cells (NESCs)	(1) 1, 5, 10, 20 and 40 μg/mL of curcumin (2) DMSO as vehicle 24, 48 and 72 h	-100% apoptotic cell deaths at 40 μg/mL of curcumin but < 20 μg/mL had no significant apoptotic effects on endometrial stromal cells (ESCs) -IL-6, IL-8, IP-10, G-CSF, MCP-1 and RANTES were highly expressed in EESCs -IL-10 and IL-12 expressions were not different in both EESCs and NESCs -Curcumin treatment significantly inhibited secretion of IL-6, IL-8, IP-10, G-CSF, MCP-1 and RANTES in EESCs after 48 h -Curcumin significantly promoted IL-10 and IL-12 secretion in EESCs after 48 h -IL-17 was completely absent in the media after treatment with curcumin for 24 and 48 h -Curcumin inhibited the phosphorylation of IKKα, IKKβ and NF-κB in EESCs -Curcumin treatment significantly inhibited the phosphorylation of JNK and STAT3 in EESCs -JNK expression significantly decreased after curcumin treatment	[83]
Bovine cumulus–oocyte complex cultured in a TCM 199 plus peritoneal fluid of infertile women with endometriosis	(1) TCM199 (control) (2) TCM199 + peritoneal fluid (PF) (3) TCM199 + PF + 0.2 mL curcumin 24 h	-GDF-9 and Kit Ligand expressions were higher in the treated group than in the non-treated group but decreased compared with the control group -TNFα expression in bovine COC cultured in PF from infertile women with endometriosis was reduced compared with those in the non-treated group -TNF-α was absent in the control group	[84]
Endometriotic stromal cells, normal endometrial stromal cells, endometriotic epithelial cells and normal endometrial epithelial cells	10, 30 and 50 μM of curcumin (2) 10 μL of WST-8 0, 24, 48, 72 and 96 h of curcumin treatment Oestradiol (E2) assay: 24 h incubation period ELISA: WST-8 for 4 h	-E2 value of endometriotic epithelial cells was higher than the endometriotic stromal cells -Expression of E2 in normal endometrial stromal and epithelial cells was extremely low -WST-8 result showed that compared with endometrial stromal cells, ectopic endometriotic stromal cells had a higher growth rate -The number of endometriotic stromal cells was reduced, and cell growth slowed compared with the 0 μmol/L group after curcumin treatment -Compared with the 0 μmol/L group, E2 level was lower after treatment with curcumin, especially in the 30 and 50 μmol/L groups	[85]
Primary cell culture of human endometriotic stromal cells from women with endometriosis and eutopic endometrial stromal cells with no endometriosis	0, 20 and 50 μmol/l of curcumin 48 h 72 h (for immunostaining assay)	-Cell morphology between ectopic and eutopic stromal cells was similar (spindle-shaped, abundant cytoplasm and oval-shaped nucleus); positive vimentin biomarker -Altered morphology, reduced permeability and increased cell suspension at 20 μmol/l curcumin after 48 h -Adherent cell decreased; a significant increase in cell suspension and presence of cell debris at 50 μmol/l curcumin after 48 h -Curcumin decreased the eutopic and ectopic cell growth -At 20 and 50 μmol/l curcumin after 48 h, endometriotic and endometrial stromal cells demonstrated increased percentages of G1-phase cells and decreased percentages of S-phase cells -Treatment with 20 and 50 μmol/l curcumin decreased the proportion of VEGF positive expression compared with the 0 μmol/l group -More in late apoptosis than early apoptosis in both endometriotic and endometrial cells	[86]

Abbreviation: TNF-α: tumor necrosis factor-α; ICAM-1: intracellular adhesion molecule-1; VCAM-1: vascular cell adhesion protein-1; IL-6: interleukin-6; IL-8: interleukin-8; MCP-1: monocyte chemoattractant protein-1; NF-κB: nuclear factor κ-light-chain-enhancer of activated B; IκB: inhibitor nuclear factor of kappa B; IP-10: interferon gamma induced protein 10; G-CSF: granulocyte colony stimulating factor; IKKα/β: inhibitor of nuclear factor κ-B kinase subunit α/β; RANTES: regulated upon activation, normal T cell expressed and presumably secreted; JNK: c-Jun N-terminal kinases; STAT3: signal transducer and activator of transcription 3; TCM199: tissue culture medium 199; GDF-9: growth differentiation factor-9; COC: cumulus-oocyte complex; WST-8: cell counting kit 8.

## Data Availability

Not applicable.

## References

[1] Dosoky N.S., Setzer W.N. (2018). Chemical Composition and Biological Activities of Essential Oils of Curcuma Species. Nutrients.

[2] Sharifi-Rad J., Rayess Y.E., Rizk A.A., Sadaka C., Zgheib R., Zam W., Sestito S., Rapposelli S., Neffe-Skocińska K., Zielińska D. (2020). Turmeric and Its Major Compound Curcumin on Health: Bioactive Effects and Safety Profiles for Food, Pharmaceutical, Biotechnological and Medicinal Applications. Front. Pharmacol..

[3] Prasad S., Aggarwal B.B., Benzie I.F.F., Wachtel-Galor S. (2011). Turmeric, the Golden Spice: From Traditional Medicine to Modern Medicine. Herbal Medicine: Biomolecular and Clinical Aspects.

[4] Priyadarsini K.I. (2014). The chemistry of curcumin: From extraction to therapeutic agent. Molecules.

[5] Akram M., Afzal A., Khan U., Abdul H., Mohiuddin E., Asif M. (2010). *Curcuma longa* and Curcumin: A review article. Rom. J. Biol.-Plant Biol..

[6] Tabrizi R., Vakili S., Akbari M., Mirhosseini N., Lankarani K.B., Rahimi M., Mobini M., Jafarnejad S., Vahedpoor Z., Asemi Z. (2019). The effects of curcumin-containing supplements on biomarkers of inflammation and oxidative stress: A systematic review and meta-analysis of randomized controlled trials. Phytother. Res..

[7] Gómez-Estaca J., Balaguer M., López-Carballo G., Gavara R., Hernández-Muñoz P. (2017). Improving antioxidant and antimicrobial properties of curcumin by means of encapsulation in gelatin through electrohydrodynamic atomization. Food Hydrocoll..

[8] Amalraj A., Varma K., Jacob J., Divya C., Kunnumakkara A.B., Stohs S.J., Gopi S. (2017). A Novel Highly Bioavailable Curcumin Formulation Improves Symptoms and Diagnostic Indicators in Rheumatoid Arthritis Patients: A Randomized, Double-Blind, Placebo-Controlled, Two-Dose, Three-Arm, and Parallel-Group Study. J. Med. Food.

[9] Wang M., Jiang S., Zhou L., Yu F., Ding H., Li P., Zhou M., Wang K. (2019). Potential Mechanisms of Action of Curcumin for Cancer Prevention: Focus on Cellular Signaling Pathways and miRNAs. Int. J. Biol. Sci..

[10] Ng Z.Y., Wong J.Y., Panneerselvam J., Madheswaran T., Kumar P., Pillay V., Hsu A., Hansbro N., Bebawy M., Wark P. (2018). Assessing the potential of liposomes loaded with curcumin as a therapeutic intervention in asthma. Colloids Surf. B Biointerfaces.

[11] Yang Q.-Q., Farha A.K., Kim G., Gul K., Gan R.-Y., Corke H. (2020). Antimicrobial and anticancer applications and related mechanisms of curcumin-mediated photodynamic treatments. Trends Food Sci. Technol..

[12] Song L., Zhang F., Yu J., Wei C., Han Q., Meng X. (2020). Antifungal effect and possible mechanism of curcumin mediated photodynamic technology against *Penicillium expansum*. Postharvest Biol. Technol..

[13] Thimmulappa R.K., Mudnakudu-Nagaraju K.K., Shivamallu C., Subramaniam K.J.T., Radhakrishnan A., Bhojraj S., Kuppusamy G. (2021). Antiviral and immunomodulatory activity of curcumin: A case for prophylactic therapy for COVID-19. Heliyon.

[14] Ak T., Gülçin I. (2008). Antioxidant and radical scavenging properties of curcumin. Chem. Biol. Interact..

[15] Sahebkar A., Serban M.-C., Ursoniu S., Banach M. (2015). Effect of curcuminoids on oxidative stress: A systematic review and meta-analysis of randomized controlled trials. J. Funct. Foods.

[16] Anthwal A., Thakur B.K., Rawat M.S., Rawat D.S., Tyagi A.K., Aggarwal B.B. (2014). Synthesis, characterization and in vitro anticancer activity of C-5 curcumin analogues with potential to inhibit TNF-α-induced NF-κB activation. BioMed Res. Int..

[17] Rathore S., Siddiqui M., Sharma P., Devi S., Nagar J., Khalid M. (2020). Curcumin: A Review for Health Benefits. Int. J. Sci. Res. (IJSR).

[18] González-Ramos R., Van Langendonckt A., Defrère S., Lousse J.C., Colette S., Devoto L., Donnez J. (2010). Involvement of the nuclear factor-κB pathway in the pathogenesis of endometriosis. Fertil. Steril..

[19] Gupta S.C., Patchva S., Aggarwal B.B. (2013). Therapeutic roles of curcumin: Lessons learned from clinical trials. AAPS J..

[20] Hewlings S.J., Kalman D.S. (2017). Curcumin: A Review of Its Effects on Human Health. Foods.

[21] Kocaadam B., Şanlier N. (2017). Curcumin, an active component of turmeric (*Curcuma longa*), and its effects on health. Crit. Rev. Food Sci. Nutr..

[22] Basnet P., Skalko-Basnet N. (2011). Curcumin: An anti-inflammatory molecule from a curry spice on the path to cancer treatment. Molecules.

[23] Vallée A., Lecarpentier Y. (2020). Curcumin and Endometriosis. Int. J. Mol. Sci..

[24] Prasad S., Tyagi A.K., Aggarwal B.B. (2014). Recent developments in delivery, bioavailability, absorption and metabolism of curcumin: The golden pigment from golden spice. Cancer Res. Treat..

[25] Shehzad A., Wahid F., Lee Y.S. (2010). Curcumin in cancer chemoprevention: Molecular targets, pharmacokinetics, bioavailability, and clinical trials. Arch. Pharm..

[26] Karthikeyan A., Senthil N., Min T. (2020). Nanocurcumin: A Promising Candidate for Therapeutic Applications. Front. Pharmacol..

[27] Stohs S.J., Chen O., Ray S.D., Ji J., Bucci L.R., Preuss H.G. (2020). Highly Bioavailable Forms of Curcumin and Promising Avenues for Curcumin-Based Research and Application: A Review. Molecules.

[28] Shoba G., Joy D., Joseph T., Majeed M., Rajendran R., Srinivas P.S. (1998). Influence of piperine on the pharmacokinetics of curcumin in animals and human volunteers. Planta Med..

[29] Kanai M., Imaizumi A., Otsuka Y., Sasaki H., Hashiguchi M., Tsujiko K., Matsumoto S., Ishiguro H., Chiba T. (2012). Dose-escalation and pharmacokinetic study of nanoparticle curcumin, a potential anticancer agent with improved bioavailability, in healthy human volunteers. Cancer Chemother. Pharmacol..

[30] Xu H., Gong Z., Zhou S., Yang S., Wang D., Chen X., Wu J., Liu L., Zhong S., Zhao J. (2018). Liposomal Curcumin Targeting Endometrial Cancer Through the NF-κB Pathway. Cell. Physiol. Biochem..

[31] Teede H., Deeks A., Moran L. (2010). Polycystic ovary syndrome: A complex condition with psychological, reproductive and metabolic manifestations that impacts on health across the lifespan. BMC Med..

[32] Escobar-Morreale H.F. (2018). Polycystic ovary syndrome: Definition, aetiology, diagnosis and treatment. Nat. Rev. Endocrinol..

[33] Khadilkar S.S. (2019). Can Polycystic Ovarian Syndrome be cured? Unfolding the Concept of Secondary Polycystic Ovarian Syndrome!. J. Obstet. Gynaecol. India.

[34] Dennett C.C., Simon J. (2015). The role of polycystic ovary syndrome in reproductive and metabolic health: Overview and approaches for treatment. Diabetes Spectr..

[35] Kwon C.-Y., Cho I.-H., Park K.S. (2020). Therapeutic Effects and Mechanisms of Herbal Medicines for Treating Polycystic Ovary Syndrome: A Review. Front. Pharmacol..

[36] Arentz S., Smith C.A., Abbott J., Bensoussan A. (2017). Nutritional supplements and herbal medicines for women with polycystic ovary syndrome; a systematic review and meta-analysis. BMC Complement. Altern. Med..

[37] Moini Jazani A., Nasimi Doost Azgomi H., Nasimi Doost Azgomi A., Nasimi Doost Azgomi R. (2019). A comprehensive review of clinical studies with herbal medicine on polycystic ovary syndrome (PCOS). Daru.

[38] Kamal D.A.M., Salamt N., Zaid S.S.M., Mokhtar M.H. (2021). Beneficial Effects of Green Tea Catechins on Female Reproductive Disorders: A Review. Molecules.

[39] Lee Y.H., Yang H., Lee S.R., Kwon S.W., Hong E.J., Lee H.W. (2018). Welsh Onion Root (*Allium fistulosum*) Restores Ovarian Functions from Letrozole Induced-Polycystic Ovary Syndrome. Nutrients.

[40] Ong M., Peng J., Jin X., Qu X. (2017). Chinese Herbal Medicine for the Optimal Management of Polycystic Ovary Syndrome. Am. J. Chin. Med..

[41] Jamilian M., Foroozanfard F., Kavossian E., Aghadavod E., Shafabakhsh R., Hoseini A., Asemi Z. (2020). Effects of curcumin on body weight, glycemic control and serum lipids in women with polycystic ovary syndrome: A randomized, double-blind, placebo-controlled trial. Clin. Nutr. ESPEN.

[42] Heshmati J., Moini A., Sepidarkish M., Morvaridzadeh M., Salehi M., Palmowski A., Mojtahedi M.F., Shidfar F. (2021). Effects of curcumin supplementation on blood glucose, insulin resistance and androgens in patients with polycystic ovary syndrome: A randomized double-blind placebo-controlled clinical trial. Phytomed. Int. J. Phytother. Phytopharm..

[43] Sohaei S., Amani R., Tarrahi M.J., Ghasemi-Tehrani H. (2019). The effects of curcumin supplementation on glycemic status, lipid profile and hs-CRP levels in overweight/obese women with polycystic ovary syndrome: A randomized, double-blind, placebo-controlled clinical trial. Complementary Ther. Med..

[44] Heshmati J., Golab F., Morvaridzadeh M., Potter E., Akbari-Fakhrabadi M., Farsi F., Tanbakooei S., Shidfar F. (2020). The effects of curcumin supplementation on oxidative stress, Sirtuin-1 and peroxisome proliferator activated receptor γ coactivator 1α gene expression in polycystic ovarian syndrome (PCOS) patients: A randomized placebo-controlled clinical trial. Diabetes Metab. Syndr..

[45] Mohammadi S., Karimzadeh Bardei L., Hojati V., Ghorbani A.G., Nabiuni M. (2017). Anti-Inflammatory Effects of Curcumin on Insulin Resistance Index, Levels of Interleukin-6, C-Reactive Protein, and Liver Histology in Polycystic Ovary Syndrome-Induced Rats. Cell J..

[46] Fatemi Abhari S.M., Khanbabaei R., Hayati Roodbari N., Parivar K., Yaghmaei P. (2020). Curcumin-loaded super-paramagnetic iron oxide nanoparticle affects on apoptotic factors expression and histological changes in a prepubertal mouse model of polycystic ovary syndrome-induced by dehydroepiandrosterone—A molecular and stereological study. Life Sci..

[47] Abuelezz N.Z., Shabana M.E., Abdel-Mageed H.M., Rashed L., Morcos G.N.B. (2020). Nanocurcumin alleviates insulin resistance and pancreatic deficits in polycystic ovary syndrome rats: Insights on PI3K/AkT/mTOR and TNF-α modulations. Life Sci..

[48] Mohammadi S., Kayedpoor P., Karimzadeh-Bardei L., Nabiuni M. (2017). The Effect of Curcumin on TNF-α, IL-6 and CRP Expression in a Model of Polycystic Ovary Syndrome as an Inflammation State. J. Reprod. Infertil..

[49] Reddy P.S., Begum N., Mutha S., Bakshi V. (2016). Beneficial effect of Curcumin in Letrozole induced polycystic ovary syndrome. Asian Pac. J. Reprod..

[50] Rosenfield R.L., Ehrmann D.A. (2016). The Pathogenesis of Polycystic Ovary Syndrome (PCOS): The Hypothesis of PCOS as Functional Ovarian Hyperandrogenism Revisited. Endocr. Rev..

[51] Burt Solorzano C.M., Beller J.P., Abshire M.Y., Collins J.S., McCartney C.R., Marshall J.C. (2012). Neuroendocrine dysfunction in polycystic ovary syndrome. Steroids.

[52] Bachmeier B.E., Mirisola V., Romeo F., Generoso L., Esposito A., Dell’Eva R., Blengio F., Killian P.H., Albini A., Pfeffer U. (2010). Reference Profile Correlation Reveals Estrogen-like Trancriptional Activity of Curcumin. Cell. Physiol. Biochem..

[53] Dumesic D.A., Oberfield S.E., Stener-Victorin E., Marshall J.C., Laven J.S., Legro R.S. (2015). Scientific Statement on the Diagnostic Criteria, Epidemiology, Pathophysiology, and Molecular Genetics of Polycystic Ovary Syndrome. Endocr. Rev..

[54] Wang F., Wang S., Zhang Z., Lin Q., Liu Y., Xiao Y., Xiao K., Wang Z. (2017). Defective insulin signaling and the protective effects of dimethyldiguanide during follicular development in the ovaries of polycystic ovary syndrome. Mol. Med. Rep..

[55] Barber T.M., Hanson P., Weickert M.O., Franks S. (2019). Obesity and Polycystic Ovary Syndrome: Implications for Pathogenesis and Novel Management Strategies. Clin. Med. Insights Reprod. Health.

[56] McCartney C.R., Marshall J.C. (2016). Polycystic Ovary Syndrome. N. Engl. J. Med..

[57] Farhood B., Mortezaee K., Goradel N.H., Khanlarkhani N., Salehi E., Nashtaei M.S., Najafi M., Sahebkar A. (2019). Curcumin as an anti-inflammatory agent: Implications to radiotherapy and chemotherapy. J. Cell. Physiol..

[58] Khashchenko E., Vysokikh M., Uvarova E., Krechetova L., Vtorushina V., Ivanets T., Volodina M., Tarasova N., Sukhanova I., Sukhikh G. (2020). Activation of Systemic Inflammation and Oxidative Stress in Adolescent Girls with Polycystic Ovary Syndrome in Combination with Metabolic Disorders and Excessive Body Weight. J. Clin. Med..

[59] Murri M., Luque-Ramírez M., Insenser M., Ojeda-Ojeda M., Escobar-Morreale H.F. (2013). Circulating markers of oxidative stress and polycystic ovary syndrome (PCOS): A systematic review and meta-analysis. Hum. Reprod. Update.

[60] Jakubczyk K., Drużga A., Katarzyna J., Skonieczna-Żydecka K. (2020). Antioxidant Potential of Curcumin—A Meta-Analysis of Randomized Clinical Trials. Antioxidants.

[61] Tanvir E.M., Hossen M.S., Hossain M.F., Afroz R., Gan S.H., Khalil M.I., Karim N. (2017). Antioxidant Properties of Popular Turmeric (*Curcuma longa*) Varieties from Bangladesh. J. Food Qual..

[62] Guo J., Cao X., Hu X., Li S., Wang J. (2020). The anti-apoptotic, antioxidant and anti-inflammatory effects of curcumin on acrylamide-induced neurotoxicity in rats. BMC Pharmacol. Toxicol..

[63] Loganes C., Lega S., Bramuzzo M., Vecchi Brumatti L., Piscianz E., Valencic E., Tommasini A., Marcuzzi A. (2017). Curcumin Anti-Apoptotic Action in a Model of Intestinal Epithelial Inflammatory Damage. Nutrients.

[64] Tomeh M.A., Hadianamrei R., Zhao X. (2019). A Review of Curcumin and Its Derivatives as Anticancer Agents. Int. J. Mol. Sci..

[65] Yan Z., Dai Y., Fu H., Zheng Y., Bao D., Yin Y., Chen Q., Nie X., Hao Q., Hou D. (2018). Curcumin exerts a protective effect against premature ovarian failure in mice. J. Mol. Endocrinol..

[66] Gozzelino R., Jeney V., Soares M.P. (2010). Mechanisms of Cell Protection by Heme Oxygenase-1. Annu. Rev. Pharmacol. Toxicol..

[67] Kádasi A., Maruniaková N., Štochmaľová A., Bauer M., Grossmann R., Harrath A.H., Kolesárová A., Sirotkin A.V. (2017). Direct effect of curcumin on porcine ovarian cell functions. Anim. Reprod. Sci..

[68] Eser A., Hizli D., Haltas H., Namuslu M., Kosus A., Kosus N., Kafali H. (2015). Effects of curcumin on ovarian ischemia-reperfusion injury in a rat model. Biomed. Rep..

[69] Behroozi-Lak T., Ebrahimpour M., Zarei L., Pourjabali M., Farhad N., Mohaddesi H. (2018). Systemic administration of curcumin nanoparticles protects ischemia-reperfusion injury in ovaries: An animal model study. Rev. Assoc. Med. Bras..

[70] Alekseyeva I.N., Makogon N.V., Bryzgina T.M., Voznesenskaya T.Y., Sukhina V.S. (2011). Effects of NF-κB blocker curcumin on oogenesis and immunocompetent organ cells in immune ovarian injury in mice. Bull. Exp. Biol. Med..

[71] Wang X.N., Zhang C.J., Diao H.L., Zhang Y. (2017). Protective Effects of Curcumin against Sodium Arsenite-induced Ovarian Oxidative Injury in a Mouse Model. Chin. Med. J..

[72] Betts D.H., Bain N.T., Madan P. (2014). The p66Shc Adaptor Protein Controls Oxidative Stress Response in Early Bovine Embryos. PLoS ONE.

[73] Kevenaar M.E., Meerasahib M.F., Kramer P., van de Lang-Born B.M.N., de Jong F.H., Groome N.P., Themmen A.P.N., Visser J.A. (2006). Serum Anti-Müllerian Hormone Levels Reflect the Size of the Primordial Follicle Pool in Mice. Endocrinology.

[74] Jana S., Paul S., Swarnakar S. (2012). Curcumin as anti-endometriotic agent: Implication of MMP-3 and intrinsic apoptotic pathway. Biochem. Pharmacol..

[75] Culley L., Law C., Hudson N., Denny E., Mitchell H., Baumgarten M., Raine-Fenning N. (2013). The social and psychological impact of endometriosis on women’s lives: A critical narrative review. Hum. Reprod. Update.

[76] Ferrero S., Evangelisti G., Barra F. (2018). Current and emerging treatment options for endometriosis. Expert Opin. Pharm..

[77] Jelodar G., Azimifar A. (2019). Evaluation of serum cancer antigen 125, resistin, leptin, homocysteine, and total antioxidant capacity in rat model of endometriosis treated with Curcumin. Physiol. Rep..

[78] Swarnakar S., Paul S. (2009). Curcumin arrests endometriosis by downregulation of matrix metalloproteinase-9 activity. Indian J. Biochem. Biophys..

[79] Zhang Y., Cao H., Hu Y.Y., Wang H., Zhang C.J. (2011). Inhibitory effect of curcumin on angiogenesis in ectopic endometrium of rats with experimental endometriosis. Int. J. Mol. Med..

[80] Kizilay G., Uz Y.H., Seren G., Ulucam E., Yilmaz A., Cukur Z., Kayisli U.A. (2017). In vivo effects of curcumin and deferoxamine in experimental endometriosis. Adv. Clin. Exp. Med..

[81] Jana S., Rudra D.S., Paul S., Snehasikta S. (2012). Curcumin delays endometriosis development by inhibiting MMP-2 activity. Indian J. Biochem. Biophys..

[82] Kim K.H., Lee E.N., Park J.K., Lee J.R., Kim J.H., Choi H.J., Kim B.S., Lee H.W., Lee K.S., Yoon S. (2012). Curcumin attenuates TNF-α-induced expression of intercellular adhesion molecule-1, vascular cell adhesion molecule-1 and proinflammatory cytokines in human endometriotic stromal cells. Phytother. Res..

[83] Chowdhury I., Banerjee S., Driss A., Xu W., Mehrabi S., Nezhat C., Sidell N., Taylor R.N., Thompson W.E. (2019). Curcumin attenuates proangiogenic and proinflammatory factors in human eutopic endometrial stromal cells through the NF-κB signaling pathway. J. Cell. Physiol..

[84] Hendarto H., Yohanes Ardianta Widyanugraha M., Widjiati W. (2018). Curcumin improves growth factors expression of bovine cumulus-oocyte complexes cultured in peritoneal fluid of women with endometriosis. Int. J. Reprod. Biomed..

[85] Zhang Y., Cao H., Yu Z., Peng H.Y., Zhang C.J. (2013). Curcumin inhibits endometriosis endometrial cells by reducing estradiol production. Iran J. Reprod. Med..

[86] Cao H., Wei Y.X., Zhou Q., Zhang Y., Guo X.P., Zhang J. (2017). Inhibitory effect of curcumin in human endometriosis endometrial cells via downregulation of vascular endothelial growth factor. Mol. Med. Rep..

[87] Bharti A.C., Donato N., Singh S., Aggarwal B.B. (2003). Curcumin (diferuloylmethane) down-regulates the constitutive activation of nuclear factor-kappa B and IkappaBalpha kinase in human multiple myeloma cells, leading to suppression of proliferation and induction of apoptosis. Blood.

[88] Liu H., Lang J.H. (2011). Is abnormal eutopic endometrium the cause of endometriosis? The role of eutopic endometrium in pathogenesis of endometriosis. Med. Sci. Monit..

[89] Laschke M.W., Menger M.D. (2018). Basic mechanisms of vascularization in endometriosis and their clinical implications. Hum. Reprod. Update.

[90] Meyer M., Clauss M., Lepple-Wienhues A., Waltenberger J., Augustin H.G., Ziche M., Lanz C., Büttner M., Rziha H.J., Dehio C. (1999). A novel vascular endothelial growth factor encoded by Orf virus, VEGF-E, mediates angiogenesis via signalling through VEGFR-2 (KDR) but not VEGFR-1 (Flt-1) receptor tyrosine kinases. EMBO J.

[91] McLaren J., Prentice A., Charnock-Jones D.S., Millican S.A., Müller K.H., Sharkey A.M., Smith S.K. (1996). Vascular endothelial growth factor is produced by peritoneal fluid macrophages in endometriosis and is regulated by ovarian steroids. J. Clin. Investig..

[92] Kaczanowski S. (2016). Apoptosis: Its origin, history, maintenance and the medical implications for cancer and aging. Phys Biol..

[93] Kutscher L.M., Shaham S. (2017). Non-apoptotic cell death in animal development. Cell Death Differ..

[94] Ravindran J., Prasad S., Aggarwal B.B. (2009). Curcumin and cancer cells: How many ways can curry kill tumor cells selectively?. AAPS J..

[95] Taylor H.S., Giudice L.C., Lessey B.A., Abrao M.S., Kotarski J., Archer D.F., Diamond M.P., Surrey E., Johnson N.P., Watts N.B. (2017). Treatment of Endometriosis-Associated Pain with Elagolix, an Oral GnRH Antagonist. N. Engl. J. Med..

